# Superconducting and superionic behaviors of electride Na_6_C under moderate pressure

**DOI:** 10.1016/j.isci.2025.112103

**Published:** 2025-02-25

**Authors:** Chang Wang, Pengye Liu, Daoyuan Zhang, Yanliang Wei, Tian Cui, Zhao Liu

**Affiliations:** 1 Institute of High Pressure Physics, School of Physical Science and Technology, Ningbo University, Ningbo 315211, People's Republic of China; 2 State Key Laboratory of Superhard Materials, College of Physics, Jilin University, Changchun 130012, People's Republic of China

**Keywords:** electronic structure of bulk materials, superconductivity, electrical property

## Abstract

Electrides exhibiting diverse electride states, superconductivity, and superionic behavior have attracted considerable attention for elucidating intricate chemical bonding and particle interactions. However, due to the scarcity of electrides exhibiting three-dimensional electride states, the associated properties abovementioned remain elusive. Here, we propose an electride *C*2*/m*-Na_6_C that exhibits peculiar three-dimensional electride states under 30 GPa and maintains dynamic stability at ambient conditions. Our electron-phonon interaction calculations reveal that the *T*_c_ of 0.051 K at 30 GPa arises from the scattering Na/C *sp*-hybridized electrons by Na/C-derived low-frequency phonons, rather than three-dimensional electride states. Further molecular dynamics simulations indicate that this structure exhibits superionic states at 1,200 K, where the unusually heavy sodium atoms exhibit diffusive behavior. This anomalous behavior can be attributed to the duality of formation process of three-dimensional electride states and their multi-center bonding effect. Our study provides theoretical guidance for further investigation into the diverse physical characteristics of electrides.

## Highlights

• The 3D electride Na_6_C exhibits superconductivity, superionic behavior at 30 GPa
• The superconductivity arises from the scattering Na/C *sp*-hybridized electrons by phonons
• The heavy sodium ions exhibit diffusive behavior relative to the lightweight carbon ions
• The duality of 3D electride state and multi-center bonding influence the ions’ diffusion

## Graphical abstract

**Figure:**
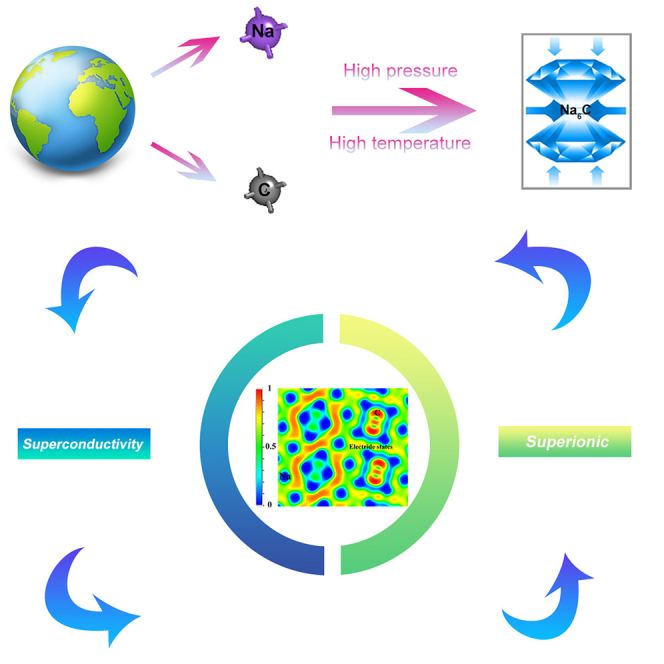

## Introduction

Electrides are a unique type of compounds wherein electrons occupy the interstitial spaces within the crystal structure and form interstitial electride states serving as anions without nuclei.^1^^,^^2^ Electrides exhibit a low work function (Φ_WF_) and high carrier mobility owing to their loosely bound nature.^3^ Due to the diverse distributions of interstitial electrons imparting a distinct character to these materials, they have promising application prospects in catalytic,^4^ electron emitters,^5^ organic light emitting diodes,^6^ and insulating materials,^7^ which have aroused a wide range of research interests. Based on the distribution of interstitial electride states and the connection of anion channels, the electrides can be classified into zero-dimensional (0D), one-dimensional (1D), two-dimensional (2D), and three-dimensional (3D) categories, exhibiting distinctive physical properties.^8^ In 2003, Matsuishi et al. successfully synthesized electride [Ca_24_Al_28_O_64_]^4+^(4*e*^−^), representing the pioneering achievement of 0D inorganic electrides characterized by exceptional thermal stability.^9^ Subsequently, through an extensive evolutionary structure search and experimental validation, the 1D electride Sr_5_P_3_ was successfully synthesized and experimentally verified, and the unique semiconductor characteristics were discovered.^10^ The electride states demonstrate a more dispersed distribution within the interlayer region, as exemplified by Ca_2_N, a 2D electride that has been discovered.^11^ At present stage, numerous studies illustrate the electride states character play an important role in the performance of diverse quantum state phenomena such as superconductivity. For instance, electride Li_8_Au and Li_6_P possessing the *p*-orbital-like electride states exhibit high-*T*_c_ of 73.1 K at 250 GPa and 41.36 K at 200 GPa.

Superionic state represents a unique phase of matter characterized by a lattice structure that exhibits both solid and liquid properties.^12^^,^^13^ The combination of superconducting behavior associated with electrons and superionic state relative to ions in the electrides provides a unique platform to study the intricate physical properties.^14^ Especially, the superconducting behavior in Li-based electrides has attracted wide attentions due to their high *T*_c_s, such as Li_6_As,^15^ Li_5_C^16^ and Li_6_C.^17^ And recent studies have revealed that superconducting and superionic states can exist in the same Li-based electride. The simulations have demonstrated that electride AsLi_7_ exhibits superconductivity of 38.4 K and superionic behavior when subjected to high pressure and temperature.^18^ Electride Li_6_Al has a *T*_c_ of around 29 K at 150 GPa, and it shows a superionic state behavior at 1,600 K, where the diffusion is found to be affected by electride states and atomic collective motion.^19^ Alkali metal alloys serve as a new platform for investigating the diverse physical properties and their manipulation under varying pressure and temperature conditions, offering valuable insights into fundamental scientific phenomena. These advancements indicate the research significance of investigating the impact of different dimensions of electride states on the complex physical properties of matter and underlying particle interactions governing them. Nevertheless, the investigation of coexisting 3D electride states and superconductivity within the same electride system under high pressure remains to be explored due to the scarcity in research.

The effective strategy for acquiring binary electrides with 3D electride states involves a significant electron transfer between two elements exhibiting substantial electronegativity disparities, wherein the metal element with lower electronegativity tends to function as an electron donor. Concurrently, a large number study has revealed that alkali metals can form electrides when they undergo charge transfer induced by pressures, where the *s-*block metal atoms are more prone to donating electrons to the interstitial sites within the lattice.^20^^,^^21^^,^^22^ The formations of high concentration electride states in Li_6_Al,^19^ Li_6_P,^23^ Li_6_As,^15^ Be_6_C,^24^ and Li_6_C^17^ are facilitated by a unique stoichiometry with electron-rich features and differences in element electronegativity. Given the same stoichiometric ratio, we are committed to exploring the Na_6_C system, with more high concentration valences, under high pressure and pursuing 3D electride states to elucidate superconducting and superionic behaviors.

In this work, we undertook multiple routes to predict the high-pressure structures of Na_6_C via swarm intelligence-based methodology CALYPSO combined with the evolutionary algorithm USPEX. Our extensive structure search has discovered a *C*2*/m*-Na_6_C phase with a particular 3D electride state under moderate pressures of 30 GPa, which remains dynamically stable down to ambient conditions. Further *ab initio* methods have been used to elucidate its electronic structures, bonding properties, mechanical stability, and superconductivity. The cooperative ionic and covalent bonds between the electride states and Na cations in *C*2*/m-*Na_6_C phase are the primary factor responsible for its structural dynamic stability. The electron-phonon coupling (EPC) calculations unveil that the presence of 3D electride states do not contribute to superconductivity, resulting in the *T*_c_ of only 0.051 K at 30 GPa. The molecular dynamics simulations indicate that this *C*2/*m* structure exhibits superionic states at high temperature of 1,200 K. The calculated lowest Φ_WF_ of *C*2*/m*-Na_6_C is 2.82 eV at ambient conditions, indicating that the electride Na_6_C with 3D electronic state holds significant potential as a catalytic material. This work is crucial for understanding the complex physical properties of matter and underlying particle interactions governing them under high pressure.

### Computation methods and details

The USPEX^25^^,^^26^ and CALYPSO codes^27^^,^^28^^,^^29^ are used to predict the crystal structures of Na_6_C within the pressure range of 0–60 GPa. Structural optimization, electron localization function (ELF) analysis,^30^ band structure calculation, and molecular dynamics simulation are all carried out with the Vienna *ab initio* Simulation Package.^31^ We further calculated the structural optimization through DS-PAW software.^32^ The Perdew-Burke-Ernzerh of (PBE) implementation of the generalized gradient approximation (GGA) is employed as exchange-correlated functional.^33^^,^^34^ The electron-ion interaction was described by the projected augmented wave pseudo-potential (PAW) approach, where the valence electrons of Na and C are represented by the numbers 2*s*^2^2*p*^6^3*s*^1^ and 2*s*^2^2*p*^2^, respectively.^35^ The Monkhorst-Pack^36^ scheme with a cut-off energy of 800 eV and the force convergence criteria were −10^−4^ eV/Å. The LOBSTER package^37^ was utilized to analyze the interatomic interaction by calculating the crystal orbital Hamilton populations (COHP)^38^ and the Integrated crystal orbital Hamilton populations (ICOHP). The phonon dispersion function calculations were implemented in the PHONOPY code,^39^ and the mechanical stability of the structure is confirmed by calculating the elastic constant, whereas the bulk modulus (B) originated from the Voigt-Reuss-Hill approximation.^40^ Originating from the result of convergence tests, *ab initio* molecular dynamics (AIMD) *NPT* simulations are carried out for 10,000 steps with a step size of 1 *fs* at the specified temperature T = 300, 1,200, and 1,500 K, respectively, for a 2 × 2 × 1 supercell with 168 atoms. The data were postprocessed using MD analysis programs.^41^ The work function (Φ_WF_) is calculated by the following formula:

$$\Phi_{\text{WF}}=E_{v}-E_{F}$$

where E_v_ denotes the vacuum level and E_F_ is the Fermi level. To compute the EPC properties, the QUANTUM ESPRESSO package was utilized within the density functional perturbation theory (DFT).^42^ In addition, the superconductivity of Na_6_C was evaluated via the McMillan formula^43^^,^^44^:

$$T_{c}=\frac{\omega_{\log}}{1.2}\exp\left(\frac{-1.04\left(1+\lambda \right)}{\lambda -\mu^{*}\left(1+0.62\lambda \right)}\right)$$

Here, $\omega_{\log}$ is the logarithmic average phonon frequency, *μ*^∗^ is the Coulomb pseudopotential, and λ is the total EPC parameter. The total EPC constant λ can be obtained by integrating the Eliashberg spectral function $\alpha^{2}F\left(\omega \right)$ as follows:

$$\lambda =2\int d\omega \frac{\alpha^{2}F\left(\omega \right)}{\omega }$$

## Results and discussion

### Crystal structure

**Figure 1:**
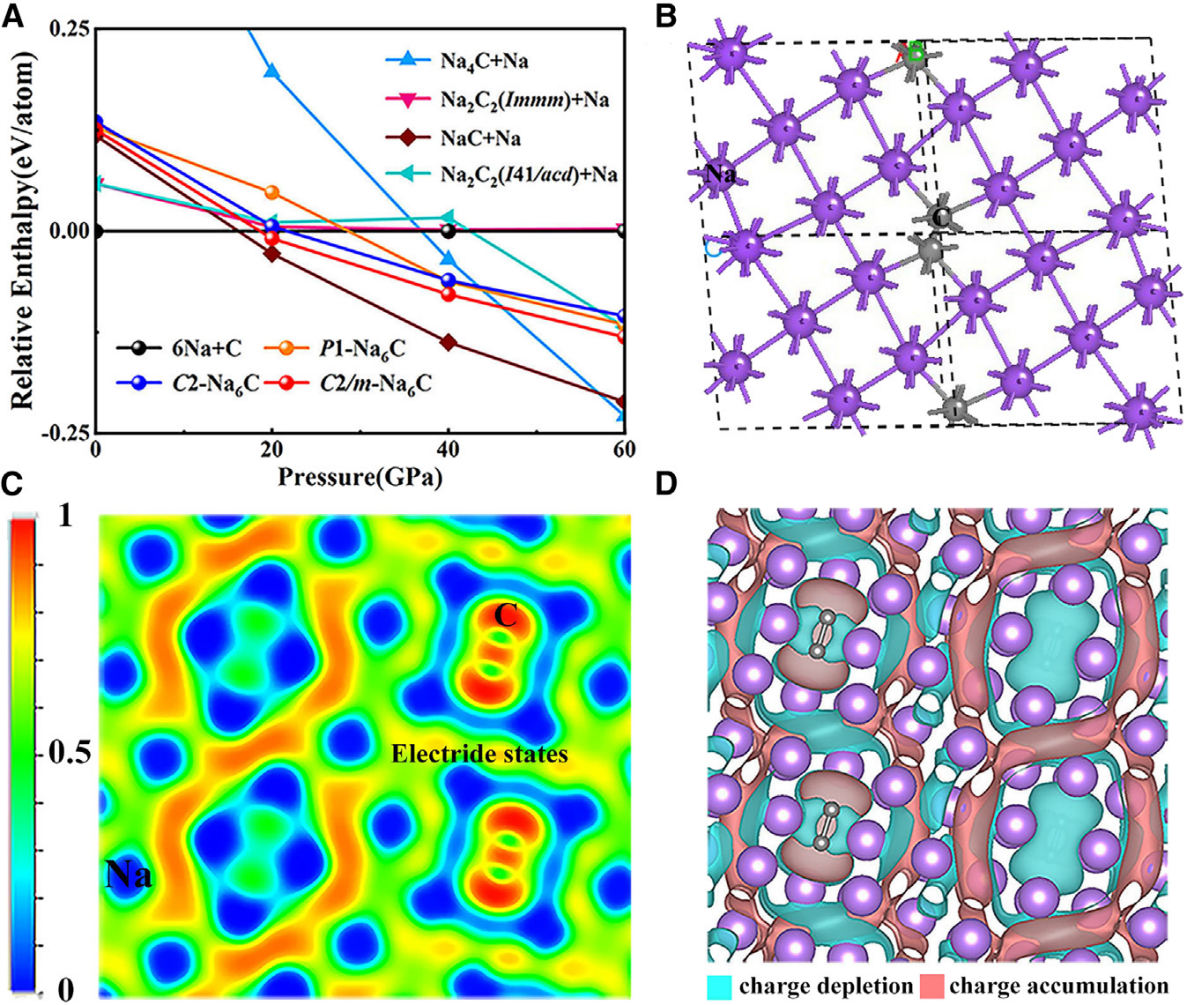
Thermodynamic stability and crystal structure of electride Na_6_C (A) The calculated enthalpy difference curves relative to 6Na + C as a function of pressures. (B) The illustration shows the structure of *C*2*/m*-Na_6_C at 30 GPa, wherein the Na atom is represented by a purple sphere and the C atom by a gray sphere. (C) 2D ELF map for (010) plane. (D) The differential charge density with an isosurface value of 0.0002 *e*/Bohr.^3^

The enthalpies of these newly predicted high-pressure phases in relation to the elements Na and C have been depicted as a pressure-phase diagram in Figure 1A. The formation enthalpy of production for per atom, Δ*H*(Na_6_C), was determined using the formula [*H*(Na_6_C) – 6∗*H*(Na) – *H*(C)]/7. Meanwhile, we calculated the formation enthalpy with respect to the potential compounds, such as NaC+5Na, 1/2Na_2_C_2_+5Na, and Na_4_C+2Na.^45^ The *C*2*/m*-Na_6_C structure has higher enthalpy than NaC+5Na in the pressure range of 0–60 GPa, indicating that it is a metastable phase. But, the enthalpy difference ΔH between them is determined to be less than 67 ± 2 meV/atom, falling within the range observed for approximately 90% of metastable materials in accordance with data from the Inorganic Crystal Structure Database.^46^ This result suggests a promising potential for synthesizing this phase. The predicted structure is shown in Figure 1B, where the *C*2*/m-*Na_6_C possess lattice parameters *a* = 8.66990 Å, *b* = 8.66990 Å, c = 7.05180 Å, and more detailed structure information is provided in Table S1. To identify whether Na_6_C is an electride and its dimensional characteristics of electride states, we then explored the electronic distribution and bonding characteristics using ELF, as shown in Figures 1C and S1. Na_6_C can be classified as a 3D electride due to the interstitial electrons being connected in the 3D interstitial space. We performed calculations on the differential charge density to better understand the formation of 3D electride states. In Figure 1D, the pink color denotes charge accumulation while the blue color represents charge depletion. The analysis results indicate an anomalous charge transfer, wherein the valence electrons of sodium and carbon atomic orbitals migrate toward the interstitial region resulting in the emergence of 3D eleectride states (will be illustrated in Figure S3 for further clarification).

### Dynamic stability

**Figure 2:**
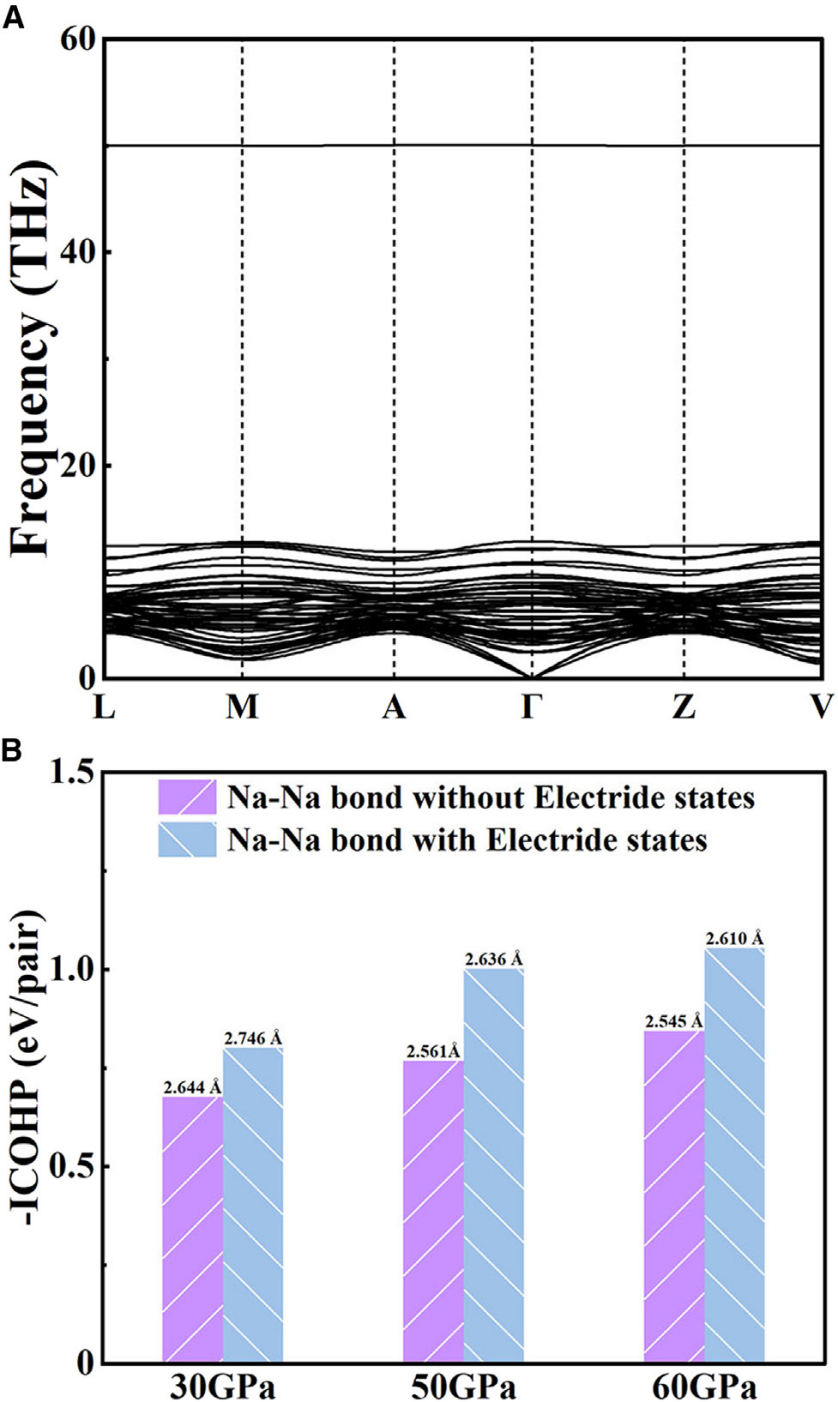
Lattice dynamics stability and bonding strength (A) Phonon dispersion curves at 30 GPa. (B) Bonding strengths with or without electride states under different pressures.

In Figure 2A, the calculated phonon dispersion indicates that the absence of imaginary frequencies throughout the entire Brillouin zone confirms the dynamic stability of *C*2*/m*-Na_6_C phase at the moderate pressure of 30 GPa. Further calculation illustrates that it exhibits noteworthy dynamic stability under ambient conditions, where the confirmation is substantiated by the phonon calculation presented in Figure S2. In order to reveal the critical impact of electride states on the structural stability, we investigated the bonding strengths between Na−Na atoms with and without electride states to determine whether the 3D electride states contribute to lowering the pressure needed for dynamic stability. With the existence of electride states, the ICOHP values for the bonding strength between Na−Na atoms with 3D electride states are ˗0.80 eV/pairs, ˗1.00 eV/pairs, and ˗1.05 eV/pairs corresponding to 30, 50, and 60 GPa, respectively, which are greater than that of bonding strengths of ˗0.68 eV/pairs, ˗0.78 eV/pairs, ˗0.84 eV/pairs without electride states as shown in Figure 2B. According to that the enthalpy (*H* = *E* + *PV*) is determined by taking the product of pressure and volume (*PV*) and plus the internal energy (*E*). The electride states facilitate the formation of covalent bonds between Na cations, resulting in lattice compression and volume reduction. In addition, the introduction of the previously mentioned electride states greatly increases the bond energy, thereby further being beneficial in reducing the energy of the system.^47^ The synergistic contributions of electride states lead to a lower enthalpy, thereby reducing the pressure required for maintaining lattice stability. The abovementioned findings suggest that the main cause of the structural dynamic stability of the *C*2*/m*-Na_6_C phase lies in the cooperative ionic and covalent interactions between electride states and Na cations.

To understand the mechanical properties and stability of electride *C*2*/m*-Na_6_C under high pressure, the elastic constants and bulk modulus have been revealed in Table S3. The mechanical stability of a crystal necessitates the strain energy to be positive, thereby implying that the entire set of elastic constants *C*_*ij*_ must satisfy the Born-Huang criterion.^48^^,^^49^ According to the elastic constants fulfills the stability criteria in Table S3, suggesting that *C*2*/m*-Na_6_C is mechanically stable under pressure. Compared with Na_5_S_3_ possessing maximum bulk modulus (B) 36 GPa among the known Na−S compounds,^50^ the electride *C*2/*m*-Na_6_C exhibits a larger B of 73.98 GPa, suggesting it has potential deformation resistance and small volume change in strain, which may be a good electrode material.

### Electronic structures and superconductivity

**Figure 3:**
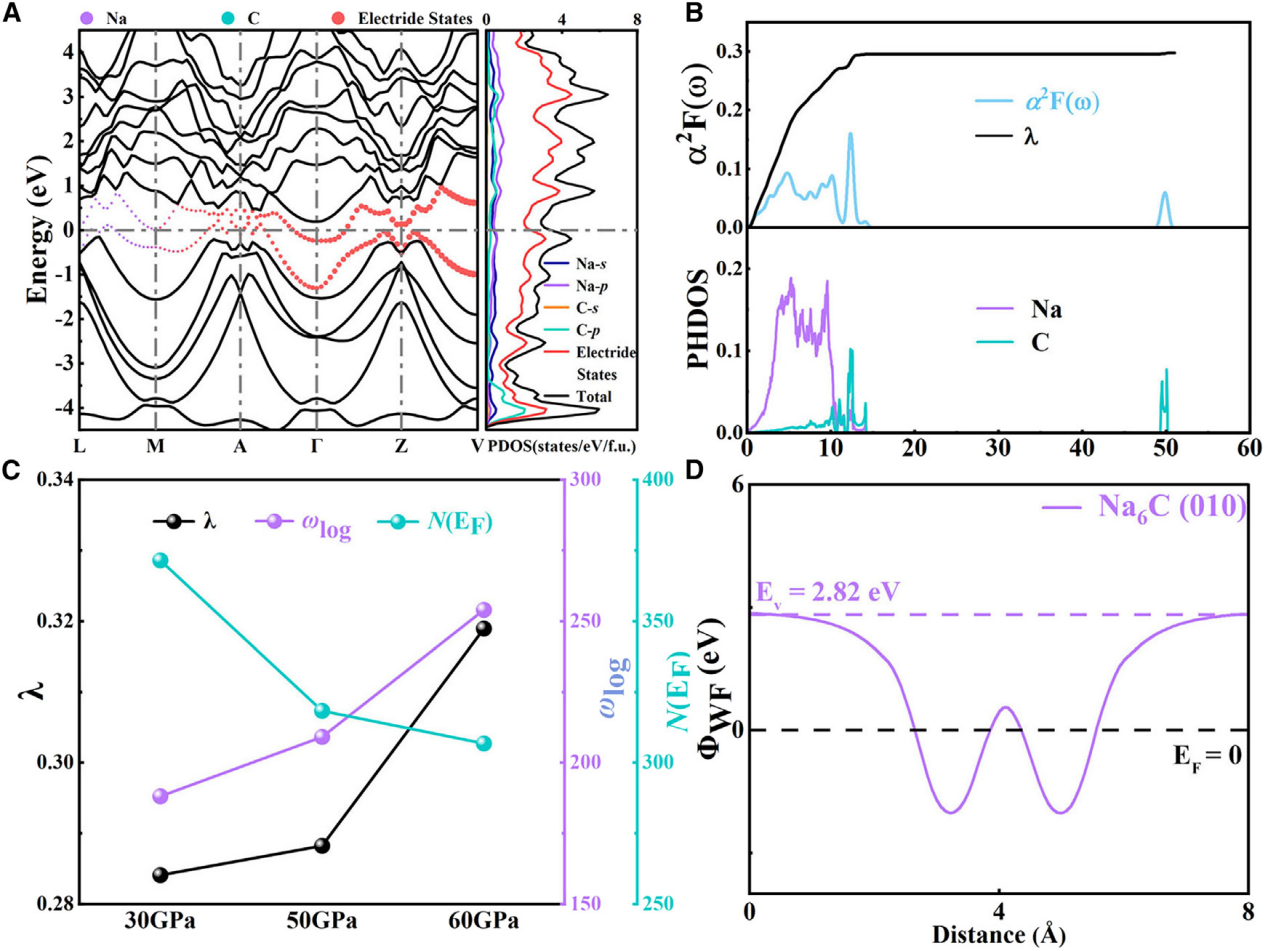
Electronic structure and superconducting properties (A) Projected band structure and density of states (DOS) at 30 GPa. The contribution of atoms to the band structure is highlighted by different colored circles. (B) The Eliashberg spectral function *α*^2^F(ω) with integrated EPC parameter λ and the phonon density of states (PHDOS) at 30 GPa. (C) The superconducting parameters at different pressures. (D) The Φ_WF_ of *C*2*/m* Na_6_C for the (010) surface at ambient condition.

Next, we calculate the electronic band structure at 30 GPa to reveal its electronic structure characteristics. The metallic properties of Na_6_C are evident due to the presence of two energy bands that cross the Fermi level. The formation of metallic states primarily arises from the contribution of 3D electride states, which occupy an *N*(E_F_) ratio of 69% at the E_F_, as depicted in Table S4. And the Na and C atoms occupy a small proportion on the Fermi surface, further indicating that a lot of valence electron transfer to the lattice cavity region. As illustrated in Figures 3A and S4, we notice that the bands dispersion of Na-*s*, Na-*p*, and C-*p* orbitals remains similar throughout the contour profile of density of states (DOS), signifying the occurrence of *sp*-orbital hybridization. Considering the significant population of electrons in close proximity to the E_F_, we explore its superconductivity through the EPC calculations using McMillan formula with a typical Coulomb pseudopotential parameter *μ*^∗^ of 0.1, where the EPC parameter λ is 0.28 and the *T*_c_ of the *C*2*/m*-Na_6_C phase at 30 GPa is about 0.051 K.^44^ Contrary to expectations, we obtained a relatively weak *T*_c_, which prompted us to further investigate the potential effects of 3D electride states on superconductivity. The Eliashberg spectral function *α*^2^*F*(*ω*) with integrated EPC parameter λ and PHDOS are displayed in Figure 3B. The PHDOS associated with Na atoms being concentrated at a lower frequency than the C atom due to its larger atomic mas. By combining the PHDOS with the Eliashberg spectral function, it can be inferred that the EPC interaction is predominantly governed by low-frequency phonons (0−15 THz), which arise from coordinated vibrations between Na and C atoms, accounting for 98% of the total λ. Conversely, the contribution to superconductivity from phonons primarily associated with stretching vibrations between C_2_ units (∼50 THz) is found to be negligible.

To deepen insight into the superconducting response to pressure of Na_6_C, we further explored the evolution of *T*_c_ with pressurization. We examined the EPC parameter λ, the electronic DOS at E_F_ [*N*(E_F_)], and the logarithmic average phonon frequency at various pressures, as shown in Figure 3C. At a pressure of 60 GPa, the EPC parameter λ becomes 0.32, yielding a *T*_c_ of 0.216 K. Analyzing the pressure-dependent λ and *ω*_log_, it is evident that λ and *ω*_log_ play a primary role in the evolution of *T*_c_ with pressure. The λ increases with pressurization, while the *N*(E_F_) decreases due to the decrease of 3D electride states at E_F_. However, there is a slight increase in the percentage of free electrons in Na and C orbitals, as shown in Figure S3A and Table S5. These results indicate that the λ is inversely proportional to the total number of interstitial electrons and proportional to the total number of free electrons in *N*(E_F_). Thus, the increase in λ is mainly attributed to the hybridized electrons occupying the host Na and C orbitals scatter by the Na/C-derived low-frequency phonons.

### Work function

Given the abundant distribution of 3D electride states in close proximity to the E_F_, we conducted simulations to assess their Φ_WF_. Generally, electrides with larger interstitial voids can induce weakly bound anionic electrons and, thus, exhibit a low Φ_WF_, which enhances their ability to donate electrons to the lowest unoccupied molecular orbital of the adsorbed molecule and reduce its activation energy. Therefore, the Φ_WF_ value of electride is considered to be an important factor affecting the catalytic performance. In Figure 3D, our calculation reveals that the Na_6_C really exhibits a low Φ_WF_, where the Φ_WF_ in the (010) plane is simulated to be 2.82 eV, which is much lower than that of intermetallic compound LaCu_0.67_Si_1.3362_ (3.50 eV) possessing a high performance for NH_3_ catalyst.^51^ Additionally, it closely resembles Y_2_C,^52^ with a Φ_WF_ of 2.84 eV and falls within the range of 2.6–3.5 eV observed for Ca_2_N electrides.^11^^,^^53^ The obtained results suggest that our proposed electride Na_6_C with 3D electronic state holds significant potential as a catalytic material.

### Superionic behaviors

**Figure 4:**
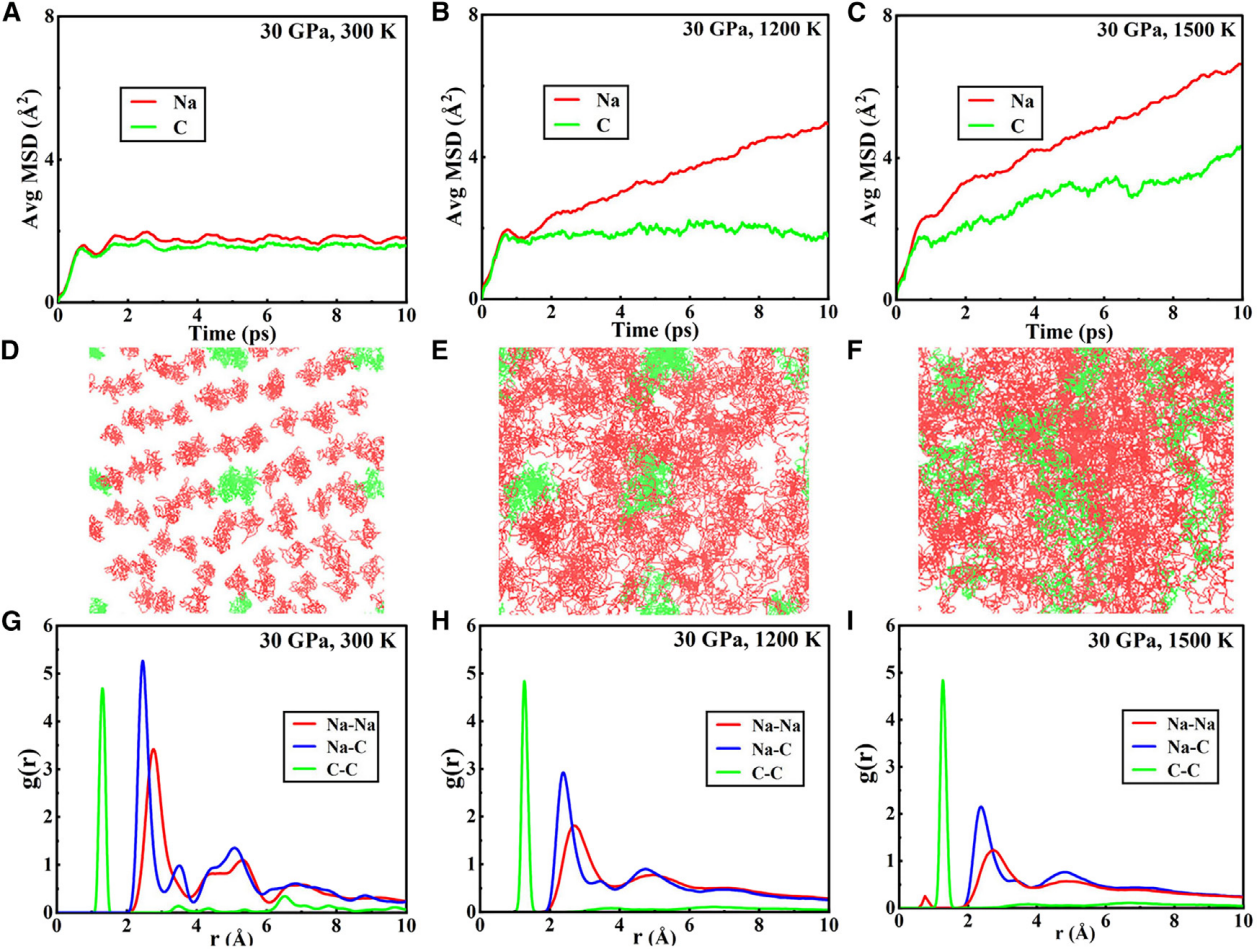
The diffusion effect of ions with temperature (A–C) The averaged mean-squared displacements (AMSD) of Na and C atoms at different temperatures within *NPT* condition. (D–F) The representations of atomic trajectories in the supercell at 300 K, 1,200 K, and 1,500 K. (G–I) Radial distribution functions (RDF) curves of Na−Na, Na−C, and C−C at different temperatures. Dynamical behaviors of Na (red) and C (green) atoms in the Na_6_C electride at 30 GPa and high temperature.

To investigate the high-temperature ionic behavior of *C*2/*m*-Na_6_C, we have performed extensive AIMD simulations within the pressure of 30 GPa and the temperature of 300 K, 1,200 K, and 1,500 K. The simulated mean-square displacements (MSDs), trajectories and radial distribution functions (RDF) curves are presented in Figure 4. The MSD slopes for all the atoms are zero at 300 K (*D*_Na_ = 0, *D*_C_ = 0), which means that the atoms stay near their equilibrium positions. Meanwhile, the trajectory diagram in Figure 4D shows that the atoms remain in the solid phase at this temperature, which indicates that they can survive at room temperature. We further simulated the kinetic behavior of Na_6_C at high temperatures of 1,200 K and 1,500 K. At 1,200 K, we found that carbon atoms oscillate around their equilibrium positions, while sodium atoms leave their equilibrium positions and become diffusive, with a diffusion coefficient of 3.918 × 10^−5^ cm^2^/s, which is the typical superionic behavior, as described in Figures 4B and 4E. Additionally, the phase transitions can also be justified by the RDF *g*(r) of Na−Na, Na−C, and C−C (see Figures 4G–4I). At 300 K, the RDF of solid Na_6_C exhibits distinct and well-defined peaks; however, with increasing temperature, these peaks gradually diminish in height and broaden, indicating a transition from a crystalline to an amorphous state^18^^,^^19^. Upon further increasing the temperature to 1,500 K, *D*_Na_ *>* 0 and *D*_C_ *>* 0 are observed, suggesting that sodium and carbon atoms exhibit liquid-like behavior, confirming complete system “melts”. Moreover, the trajectory diagram demonstrates unrestricted diffusion of all atoms. The relationship between the “melting points” of different elements is inferred as *T*_Na_ *< T*_C_.

The principle of equipartition governs that in a state of equilibrium, energy should be uniformly distributed among the various degrees of freedom, resulting in lighter atoms exhibiting higher velocities while possessing an equivalent amount of kinetic energy. Intriguingly, within our system, sodium atoms exhibit higher mobility and a lower “melting” temperature compared to carbon atoms, despite the fact that sodium is heavier than carbon. To elucidate the underling causation of this anomalous diffusion in view of chemical bonding strength, the 3D and 2D ELF at 30 GPa have unequivocally confirmed that formation of a strong covalent bonds between C−C (refer to Figures 1B and S1). And the calculated strength of this covalent bond for C−C at 30 GPa amounts to ˗18.06 eV/pairs, surpassing significantly the bonding strength observed between Na−Na based on ICOHP value. Consequently, two carbon atoms form an extensively condensed diatomic entity exhibiting a molecular configuration with double the atomic mass as compared to sodium atoms, thereby rendering it more stable than sodium atoms response to high temperature. To gain a deeper understanding of the pivotal role played by 3D electride states in this anomalous diffusion phenomenon, we performed calculations to ascertain the electron population up to the E_F_ for each element at 30 GPa. Our findings reveal that each carbon atom contributes 1.5 eV to the interstitial cavity, surpassing the 0.75 eV contributed by sodium atoms, as seen in Table S6. Consequently, a stronger ionic interaction is established between carbon atoms and the 3D electride states compared to sodium atoms, empowering them more stable in their equilibrium positions. As a result, carbon atoms exhibit a higher diffusion barrier in superionic states relative to sodium atoms, thereby making sodium atoms more prone to diffusion than carbon atoms. The aforementioned analyses demonstrate that the duality of formation progress of 3D electride states and their multi-center bonding effect play a pivotal role in the abnormal behavior of ions.

### Conclusions

In summary, we have successfully predicted the 3D electride states in Na_6_C electride within the moderate pressure range of 0–60 GPa and demonstrated its potential synthesizable pathways. In conjunction with first principles calculations, our proposed *C*2*/m*-Na_6_C electride exhibits multiple properties including the electride state, superconductivity, and superionic behavior under moderate pressure. The synergistic effects of ionic and covalent bonding between electride states and cations significantly influence the structural dynamic stability of the *C*2/*m*-Na_6_C phase under moderate pressures. The EPC is primarily attributed to the *sp-*hybridized electrons of Na and C atoms that were scattered by Na/C-derived low-frequency phonons, while the presence of 3D electride states do not significantly contributes to superconductivity, resulting in a modest *T*_c_ of only 0.051 K at 30 GPa. Our molecular dynamics simulations indicate that at 30 GPa and 1,200 K, the unusually heavy sodium atoms exhibit diffusive behavior to perform superionic behaviors while the lighter carbon atoms remain in lattice equilibrium positions due to the formation process of 3D electride states and their multi-center bonding effect. Research on its multifunctional potential properties indicates that the lowest work function of *C*2/*m*-Na_6_C is 2.82 eV at atmospheric pressure, indicating its significant potential as a catalytic material. Our findings advance the understanding of superconducting and superionic behaviors consisting of 3D electride states under moderate pressure and stimulate future theoretical and experimental studies in this field.

### Limitations of the study

Our reported superconducting electride *C*2/*m*-Na_6_C with 3D electride states holds significant implications for understanding complex chemical bonding and ion interactions. However, research on the superconductivity of this class of electrides remains limited due to the scarcity of this type electrides and inadequate mechanistic studies that could elucidate the role of electride states in EPC. Especially, the varying charge densities and electron concentrations within the crystal cavities, and whether they can exhibit free-electron behavior under high pressure, require further investigation. Moreover, the *T*_c_ of electrides under pressure remains significantly low. Designing new high-temperature superconducting electrides and conducting experimental characterization remain challenging tasks.

## Resource availability

### Lead contact

Further information and requests for resources should be directed to and will be fulfilled by the lead contact, Zhao Liu (liuzhao@nbu.edu.cn).

### Materials availability

This study did not generate new reagents.

### Data and code availability

• Data: All data reported in this paper will be shared by the lead contact upon request.
• Code: This paper does not report original code.
• Other: Any additional information required to reanalyze the data reported in this paper is available from the lead contact upon request.

## Acknowledgments

This work was supported by the 10.13039/501100012166National Key Research and Development Program of China (grants no. 2023YFA1406200), 10.13039/501100001809National Natural Science Foundation of China (grants no. 12304021, no. 52072188), 10.13039/501100004731Zhejiang Provincial Natural Science Foundation of China (grant no. LQ23A040004), Natural Science Foundation of Ningbo (grant no. 2022J091), Program for Science and Technology Innovation Team in Zhejiang (grant no. 2021R01004), and Program for Changjiang Scholars and Innovative Research Team in University (no. IRT_15R23). The calculations were performed in the Supercomputer Center of NBU. We gratefully acknowledge HZWTECH for providing computation facilities.

## Author contributions

C.W., writing – original draft; P.L., investigation and data curation; D.Z., investigation and data curation; Y.W., investigation; T.C., writing – review and editing and supervision; Z.L., writing – review and editing and supervision.

## Declaration of interests

The authors declare no competing interests.

## STAR★Methods

### Key resources table

**Table:** 

REAGENT or RESOURCE	SOURCE	IDENTIFIER
**Software and algorithms**		

USPEX	https://uspex-team.org/zh	N/A
CALYPSO	http://www.calypso.cn/	N/A
VASP	https://www.vasp.at/	N/A
PHONOPY	http://phonopy.github.io/phonopy/	N/A
QUANTUM ESPRESSO	https://www.quantumespresso.org	N/A
VMD	https://www.ks.uiuc.edu/Research/vmd/	N/A
VESTA	http://jp-minerals.org/vesta/en/download.html	N/A
MATERIALS STUDIO	https://www.3ds.com/products/biovia/materials-studio	N/A
ORIGIN	https://www.originlab.com/	N/A
ADOBE PHOTOSHOP	https://www.adobe.com/products/photoshop.html	N/A

### Experimental model and study participant details

This work does not use any experimental models.

### Method details

#### Specific methods

The calculations were performed using the plane-wave pseudopotential method as implemented in the QUANTUM ESPRESSO package. We set the wave function cutoff energy to 100 Ry and the charge density cutoff energy to 1000 Ry. For Brillouin zone integrations, we utilized a 16×16×8 ***k***-point mesh with Gaussian smearing of 0.03 Ry. The phonon dispersion and electron-phonon coupling (EPC) calculations were based on density functional perturbation theory (DFPT) and were performed using a 4×4×2. ***q***-point grid. And the structural optimization was further verified through DS-PAW software.

#### Electronic structure calculations and chemical bonding analysis

(1) Density Functional Theory (DFT) calculations were performed using Vienna ab initio Simulation Package
(2) The analysis of interatomic interactions and chemical bonding was conducted using the LOBSTER package.

#### Phonon calculations and electron-phonon coupling

(1) Phonon calculations were conducted using Density Functional Perturbation Theory (DFPT).
(2) The superconducting transition temperature was estimated using the Allen-Dynes McMillan- formula.

### Quantification and statistical analysis

Analyses and plots were performed with Materials Studio, VESTA, Photoshop and Origin.

### Additional resources

Additional resources, including supplementary data and detailed methodological protocols, are available upon request from the lead contact. All supplementary materials are intended to enhance the reproducibility and transparency of the study, providing comprehensive insights into the computational approaches employed. For access to these resources, please contact [lead contact, Zhao Liu (E-mail: liuzhao@nbu.edu.cn)].
