# Supplementary material for: Superconducting and superionic behaviors of electride Na6C under moderate pressure
Source: iScience. 2025 Feb 25;28(3):112103. doi: 10.1016/j.isci.2025.112103 (PMC11931384; doi:10.1016/j.isci.2025.112103)
Supplement: Document S1. Figures S1–S5 and Tables S1–S6 [file mmc1.pdf]

iScience, Volume *28*

Supplemental information

Superconducting and superionic behaviors of electride Na_6C under moderate pressure

Chang Wang, Pengye Liu, Daoyuan Zhang, Yanliang Wei, Tian Cui, and Zhao Liu

Supplementary Figures

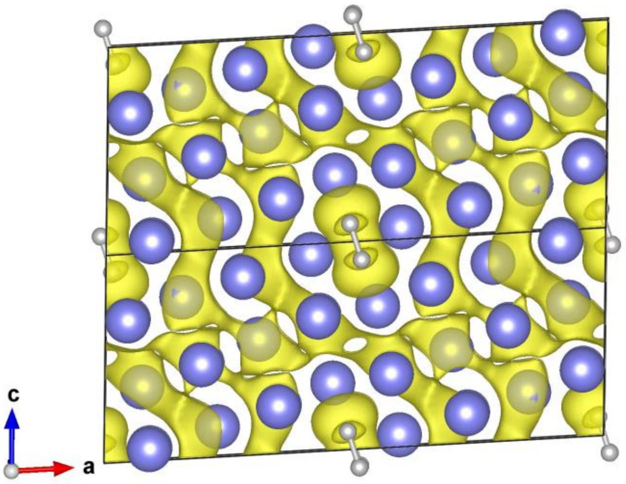

Figure S1. 3D Electron localization function of electride Na_6C, related to Figure 1. 3D ELF with isosurface of 0.65 at 30 GPa.

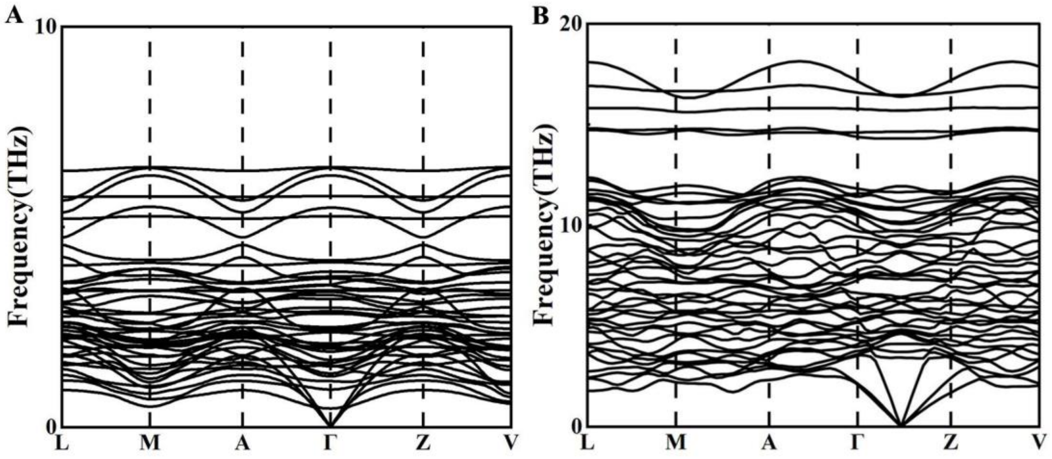

Figure S2.Lattice dynamics stability, related to Figure 2. A.)The phonon dispersion curve of *C*2/*m*-Na_6C in the 0–10 eV frequency range at atmospheric condition and B.)0–20 eV frequency range at 60 GPa.

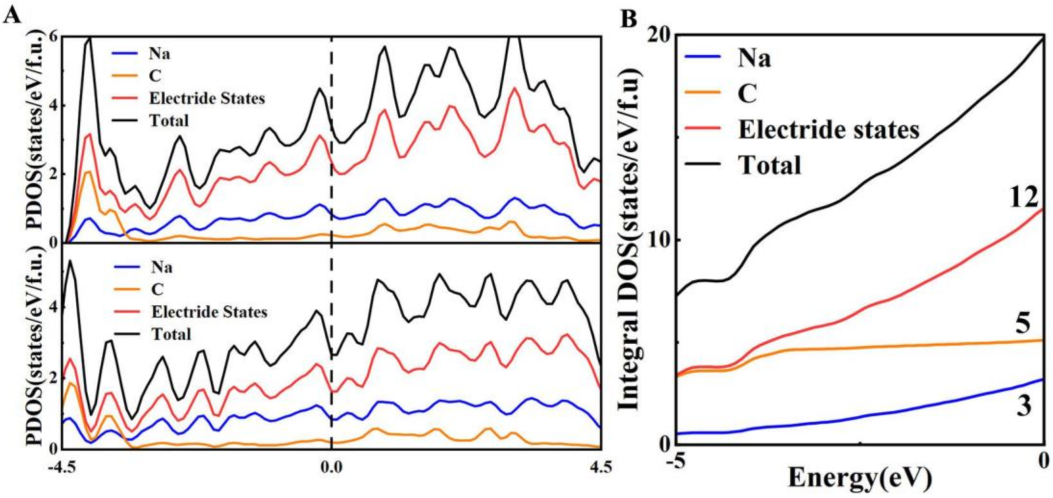

Figure S3. Electronic structure at different pressures, related to Figure 3. A.) The PDOS of *C*2/*m*-Na_6C at 30 and 60 GPa. B.) The integration electron numbers up to E_F of each element for *C*2/*m*-Na_6C phase, containing two Na_6C formula units, at 60 GPa.

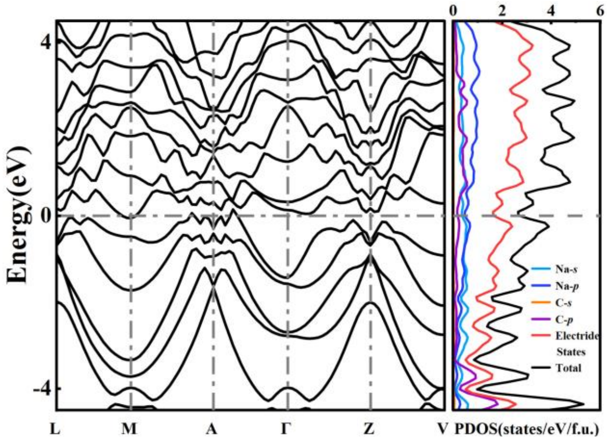

Figure S4.Electronic structures, related to Figure 3. Band structure and DOS at 60 GPa.

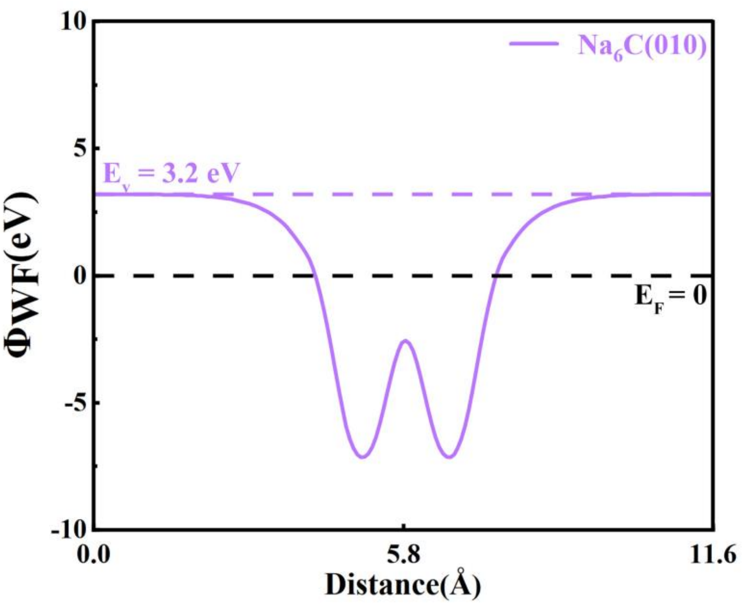

Figure S5. Work functions of different sections, related to Figure 3. Work functions of *C*2/*m* Na_6C for the (010) surface at 30 GPa.

Supplementary Table

Table S1. Crystal structure information, related to Figure 1. Structural information of the predicted Na_6C phases.

Phases	Pressure (GPa)	Lattice Parameters (Å)	Atomic coordinates (fractional)			
			Atoms	x	y	z
C*2/*m	30	*a* = 8.66990<br>*b* = 8.66990<br>*c* = 7.05180<br>*α* = 93.7324<br>*β* = 93.7324<br>*γ* = 22.4275	Na	0.57397	0.57397	0.64674
			Na	0.42603	0.42603	0.35326
			Na	0.80697	0.80697	0.4484
			Na	0.19303	0.19303	0.5516
			Na	0.65164	0.65164	0.22527
			Na	0.34836	0.34836	0.77473
			Na	0.72568	0.72568	0.85845
			Na	0.27432	0.27432	0.14155
			Na	0.04411	0.04411	0.32068
			Na	0.95589	0.95589	0.67932
			Na	0.09031	0.09031	0.94588
			Na	0.90969	0.90969	0.05412
			C	0.51189	0.51189	0.08809
			C	0.48811	0.48811	0.91191
***P*1**	40	*a* = 7.89765<br>*b* = 7.67385<br>*c* = 3.21329<br>*α* = 78.3460<br>*β* = 78.0070<br>*γ* = 90.9886	Na	-1.01694	-0.04802	-0.32054
			Na	-0.32700	-0.23966	-0.13758
			Na	-1.08448	-0.27001	-0.68323
			Na	-0.85276	-0.41985	-0.35776
			Na	-1.63150	-0.43505	-0.95035
			Na	-1.77369	-0.07832	-0.86795
			Na	-1.13838	-0.63438	-0.17329
			Na	-0.67995	-0.78527	-0.41053
			Na	-0.96227	-0.68414	-0.82847

			Na	-0.47642	-0.88224	-0.05467
			Na	-1.25087	-0.89993	-0.6468
			Na	-0.42559	-0.53281	-0.59344
			C	-0.49962	-0.23303	-0.46481
			C	-0.60834	-0.08571	-0.53883
			Na	0.65082	0.00489	0.20973
			Na	0.30849	0.13209	0.47880
			Na	0.21827	0.93165	0.08251
			Na	0.66874	0.61456	0.43540
			Na	0.06152	0.66752	0.55413
			Na	0.28480	0.52620	0.25833
***C*2**	40	a = 5.42741<br>b = 4.76767<br>c = 9.15750<br>α = 104.8882<br>β = 77.2963<br>γ = 89.4699	Na	0.89510	0.46591	0.13907
			Na	0.53590	0.34904	0.94511
			Na	0.73846	0.19345	0.61132
			Na	0.84046	0.81510	0.88266
			Na	0.12133	0.27742	0.80800
			Na	0.42328	0.74893	0.74952
			C	0.97587	0.93517	0.34654
			C	0.97658	0.20503	0.34675

Table S2. Crystal structure information, related to Figure 1. Structural information of the Na and C phases.

Phases	Pressure (GPa)	Lattice Parameters (Å)	Atomic coordinates (fractional)			
			Atoms	x	y	z
		a = 2.33856				
		b = 2.33856				
***P*1**	0	c = 3.81318	Na	0.66667	0.33333	0.75000
		α = 90.0000	Na	0.33333	0.66667	0.25000
		β = 90.0000				
		γ = 120.0000				
		a = 4.51507				
		b = 4.51505	C	0.91908	0.91902	0.91893
		c = 4.51494	C	0.24990	0.24997	0.25005
***P*1**			C	0.75009	0.75003	0.74995
	0	α = 29.7062	C	0.08092	0.08098	0.08107
		β = 29.7068				
		γ = 29.7074				

Table S3. Mechanical stability, related to Figure 2. Calculated elastic constants (C_{ij}) for Na_6C at 30 GPa, respectively. The elastic constants of the monoclinic crystal obtained by the Born-Huang theory

C_{11}	C_{22}	C_{33}	C_{44}	C_{55}	C_{66}	C_{12}
972.54	981.57	1030.64	279.24	163.35	244.04	635.30
C_{13}	C_{23}	C_{15}	C_{25}	C_{35}	C_{46}	
570.28	633.77	-23.12	-25.86	26.47	-29.84	

$$C_{11} > 0, C_{22} > 0, C_{33} > 0, C_{44} > 0, C_{55} > 0, C_{66} > 0$$

$$[C_{11} + C_{22} + C_{33} + 2(C_{12} + C_{13} + C_{23}) > 0];$$
$$(C_{33}C_{55} - C_{35}^2) > 0, (C_{44}C_{66} - C_{46}^2) > 0, (C_{22} + C_{33} - 2C_{23}) > 0;$$
$$[C_{22}(C_{33}C_{55} - C_{35}^2) + 2C_{23}C_{25}C_{35} - C_{23}^2C_{55} - C_{25}^2C_{33}] > 0;$$
$$\{2[C_{15}C_{25}(C_{33}C_{12} - C_{13}C_{23}) + C_{15}C_{35}(C_{22}C_{13} - C_{12}C_{23})$$
$$+ C_{25}C_{35}(C_{11}C_{23} - C_{12}C_{13})] - C_{15}^2(C_{22}C_{33} - C_{23}^2)$$
$$- C_{25}^2(C_{11}C_{33} - C_{13}^2) - C_{35}^2(C_{11}C_{22} - C_{12}^2) + C_{55}g\} > 0;$$
$$g = C_{11}C_{22}C_{33} - C_{11}C_{23}^2 - C_{22}C_{13}^2 - C_{33}C_{12}^2 + 2C_{12}C_{13}C_{23}$$

Table S4. Electronic structures, related to Figure 3. The proportion of different orbitals at the Fermi level.

**Proportion**	**Na-*s***	**Na-*p***	**C-*s***	**C-*p***	**Electride states**
**30 GPa**	11.4%	13%	0.4%	6.2%	69%
**60 GPa**	13.6%	17.8%	0.5%	6.9%	61.2%

Table S5. Superconducting properties, related to Figure 3. Supeconducting parameters λ, N(E$_F$), logarithmic average frequency $\omega_{\log}$ and the T_c of Na_6C at different pressures.

Pressure (GPa)	$\boldsymbol{\lambda}$	$\boldsymbol{\omega_{\log}}$	$\boldsymbol{N(E_F)}$	McMillan equation (K)
30	0.28409	188.162	371.48671	0.051
50	0.28823	209.132	318.25959	0.067
60	0.31892	253.997	306.72512	0.216

Table S6. Electronic structures, related to Figure 3. The number of electrons transferred from Na and C atoms to the interstitial space at the E$_F$.

Quantity	Fermi level	Total transfer electrons	Each atom transfers electrons
**Na**	**3**	**9**	**0.75**
**C**	**5**	**3**	**1.5**
